# KGCLMDA: a computational model for predicting latent associations of microbial drugs using knowledge graphs and contrastive learning

**DOI:** 10.1093/bioinformatics/btaf457

**Published:** 2025-08-18

**Authors:** Meiling Liu, Shujuan Su, Guohua Wang, Shan Huang

**Affiliations:** College of Computer and Control Engineering, Northeast Forestry University, Harbin, Heilongjiang, 150040, China; College of Computer and Control Engineering, Northeast Forestry University, Harbin, Heilongjiang, 150040, China; College of Computer and Control Engineering, Northeast Forestry University, Harbin, Heilongjiang, 150040, China; Department of Neurology, The Second Affiliated Hospital, Harbin Medical University, Harbin, Heilongjiang, 150081, China

## Abstract

**Motivation:**

Predicting microbe–drug associations (MDgAs) is critical for understanding the role of microbes in drug metabolism, exploring their interactions with host physiology, and advancing personalized therapy. However, traditional methods face challenges in dealing with data sparsity, information imbalance, and the extraction of complex biological knowledge, which limit the accurate prediction of microbe–drug associations. Therefore, developing a computational model that can efficiently integrate multi-source data and address the challenges of data sparsity and information imbalance is essential.

**Results:**

The paper proposes a model that integrates knowledge graphs and contrastive learning. By constructing both local and non-local association graphs, the model effectively captures the complex relationships between microbes and drugs. We preprocess and model the embedding representations of microbes and drugs, and design a multi-level interactive contrastive learning mechanism to optimize the information flow both within and outside the graph. Experimental results show that our model significantly outperforms existing methods in metrics such as AUC and AUPR, providing an efficient solution for predicting microbe–drug associations.

**Availability and implementation:**

The source code is available at: https://github.com/SJshujuan/KGCLMDA. The code used in this study is also available on Zenodo: https://doi.org/10.5281/zenodo.16754402.

## 1 Introduction

Microorganisms, among the most abundant and diverse life forms on Earth, play a critical role in human health and disease regulation (The Human Microbiome Project Consortium 2012). The human body is home to trillions of microorganisms, including bacteria, archaea, fungi, protozoa, and viruses, all of which together form the human microbiome (Gevers *et al.* 2012). These microorganisms inhabit various sites within the body and influence health in numerous ways (Sommer and Bäckhed 2013, Kumar and Chordia 2017). In particular, the microbiome and mucosal surfaces cooperate to defend against harmful pathogens (Macpherson and Harris 2004). For example, microorganisms aid in the synthesis of glycogen and the production of essential vitamins, which are crucial for immune system function (Kau *et al.* 2011). The gut microbiota is not only critical for metabolizing dietary polysaccharides but also for synthesizing essential vitamins, supporting immune development, and defending against pathogens (Durack and Lynch 2019, Zimmermann *et al.* 2019). Additionally, disruptions in the balance of microbial communities within the body can contribute to conditions such as diabetes (Wen *et al.* 2008), inflammatory bowel diseases (Durack and Lynch 2019), and cancer (Schwabe and Jobin 2013).

Various organisms, including bacteria, fungi, and plants, produce secondary metabolites, commonly referred to as natural products. These natural products have long served as an important source for drug development, with many of these compounds being developed for therapeutic use and showing significant potential for treating human diseases (Pham *et al.* 2019). Recently, as the diversity of drugs explored in medical research has expanded, microbial resistance to these drugs has become a growing concern (Zhu *et al.* 2019, Long *et al.* 2020). Traditional laboratory experiments are both expensive and time-consuming. As the issue of microbial resistance continues to escalate, there is an increasing need for efficient computational models to uncover potential microbe–drug interactions related to drug resistance.

In recent years, the rapid development of bioinformatics has led to the creation of several high-quality databases. For example, the MDAD database focuses on microbe–drug interactions, providing data on microbial sensitivity and resistance to drugs (Sun *et al.* 2018). The aBiofilm database documents the effects of antimicrobial agents on microbial biofilms, supporting research on biofilm-related infections (Rajput *et al.* 2018). The DrugVirus database covers interactions between viruses and antiviral drugs, offering resistance data (Andersen *et al.* 2020). Additionally, DRKG supports drug repositioning and disease treatment research (Ioannidis *et al.* 2022); VMH and HMDB provide research data on microbial-host metabolic networks and human metabolites, respectively (Wishart *et al.* 2018, Noronha *et al.* 2019); MPIDB specializes in microbe-protein interaction data (Goll *et al.* 2008); and NCBI offers extensive data resources for life science re-search (Wheeler *et al.* 2008). Based on these databases, researchers have developed various computational approaches to predict potential interactions between biological entities, which can be mainly categorized into four types: network-based methods, matrix factorization techniques, ma-chine learning methods, and deep learning approaches.

In the context of network-based approaches, Zhu *et al.* (2019) introduced the HMDAKATZ model, which combines microbial similarity using the GIP kernel and drug chemical structure similarity to predict microbe–drug interactions. Dong *et al.* (2023) introduced the DAEMDA model, integrating a dual-channel attention mechanism and graph neural networks to improve the prediction accuracy of miRNA-disease associations. In terms of matrix factorization methods, the MDSVDNV model designed by Tan *et al.* (2023)effectively predicts microbe–drug associations through the combination of Singular Value Decomposition (SVD) and Node2Vec, while preserving key features and reducing redundancy. Li *et al.* (2024) introduced the GSL-DTI model, which leverages automatic graph structure learning to build a drug-protein pair (DPP) network, enhancing the accuracy of drug-target interaction predictions. Regarding machine learning, Anahtar *et al.* (2021) explored its application in antibiotic resistance, but performance was limited due to the constraints of the training dataset.

Deep learning methods have demonstrated widespread application potential in the prediction field. For instance, DMGL-MDA addresses the issue of over-smoothing by combining dual-modal embedding with Graph Attention Networks (GAT) and Graph Transformers (GT); however, its scalability and computational complexity remain challenges when handling large-scale datasets (Zhu *et al.* 2024). In contrast, OGNNMDA network hierarchy through an ordered message-passing mechanism, improving embedding capacity, but still relies on known similarity matrices, which limits its adaptability to novel microbe–drug associations.(Zhao *et al.* 2024). MGAVAEMDA balances feature learning and model complexity by integrating variational autoencoders with random forests, achieving effective feature fusion; however, it fails to fully address redundancy issues in high-dimensional feature spaces (Wang *et al.* 2024). GARFMDA improves prediction interpretability and accuracy by combining graph attention networks with a bilayer random forest, but still faces high computational costs in the feature selection process (Kuang *et al.* 2024). In comparison, KGCLMDA (the model proposed in this study) introduces enhanced graph convolution and variational autoencoders, reducing model complexity while improving prediction accuracy. Notably, KGCLMDA overcomes the limitations of existing methods in handling high-dimensional data and complex microbe–drug networks, achieving superior performance across multiple evaluation metrics.

In the field of microbe–drug association prediction, particularly for anti-biotic resistance prediction, traditional methods are often constrained by data scarcity, information imbalance, and inadequate knowledge extraction. To address these challenges, we propose a model that integrates TransE preprocessing, multi-head attention, and multi-level contrastive learning. First, we use TransE to preprocess microbes and drugs, embed-ding them into a low-dimensional space to capture their latent semantic relationships. Next, we construct local graphs focusing on direct interactions between microbes and drugs, extracting first-order collaborative filtering signals from the microbe–drug interaction matrix and expanding these relationships through knowledge graph propagation. We then build non-local graphs by propagating higher-order collaborative filtering signals and external knowledge graph information to further enhance the representations of microbes and drugs. In the graph encoding phase, a multi-head attention mechanism is employed to model the relation-ships from multiple perspectives, capturing multi-level information. Finally, we apply intra- and inter-graph contrastive learning to deeply integrate direct and indirect associations, significantly improving prediction accuracy and robustness in complex, high-noise, and data-sparse conditions. Our model effectively overcomes the limitations of traditional methods, providing a novel solution for microbe–drug association prediction with significant applications in antibiotic resistance prediction.

## 2 Materials and methods

**Figure 1.**
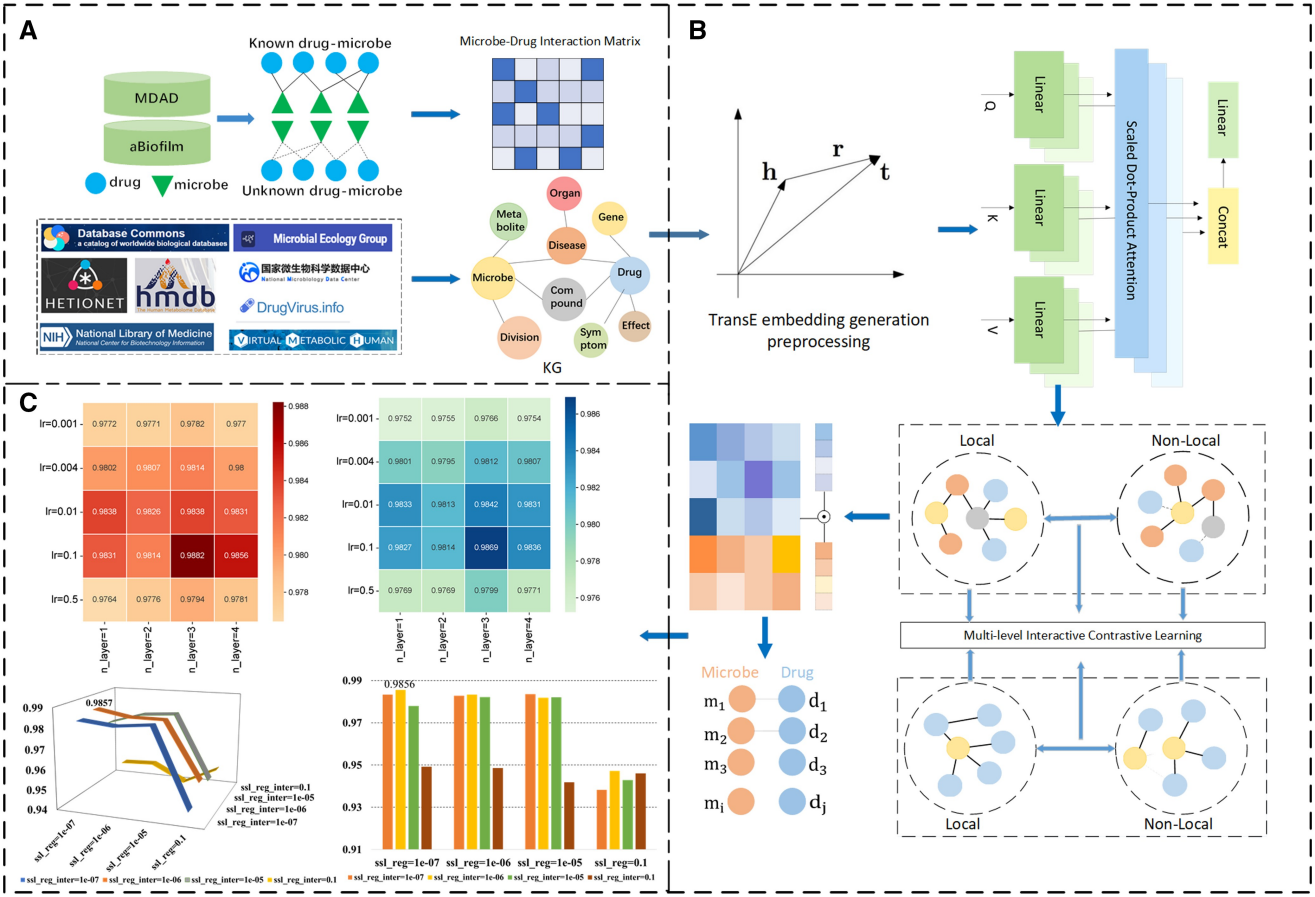
Model framework diagram. (A) Construction of the microbe–drug interaction matrix using known interactions from MDAD and aBiofilm datasets, enriched with multi-source knowledge from various databases. (B) The model architecture utilizes TransE, multi-head attention, and contrastive learning to capture both local and non-local relationships in the microbe–drug network. (C) Performance evaluation demonstrating the model’s effectiveness across varying learning rates and regularization parameters.

**Figure 2.**
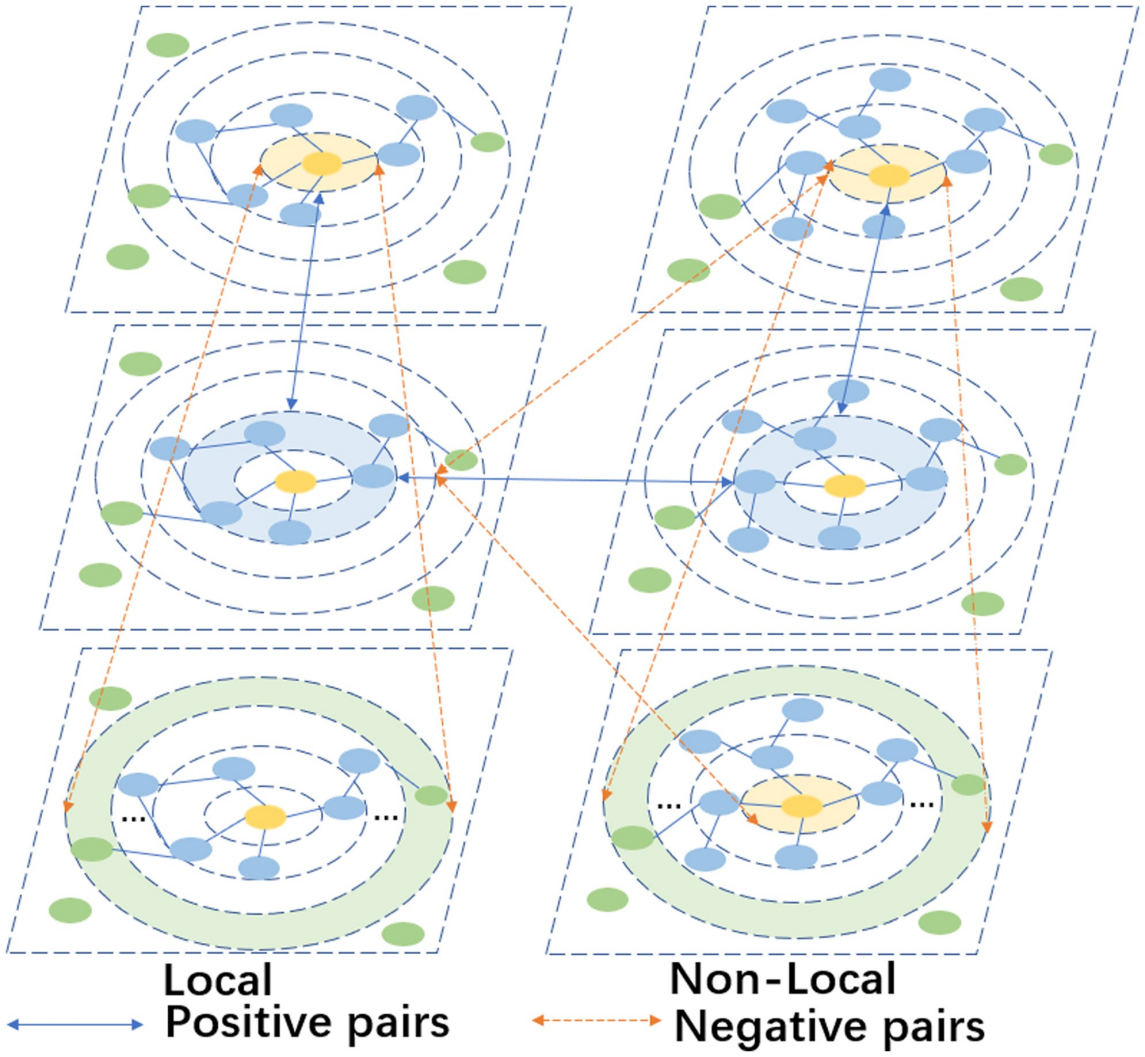
Multi-level contrastive mechanism for predicting potential microbe–drug associations. The left part is the Local module, and the right part is the Non-Local module. Arrows represent positive pairs that strengthen associations, and dashed arrows represent negative pairs that suppress interference. Entity representations are optimized through hierarchical and cross-module interactions.

We collected known microbe–drug associations from the MDAD and aBiofilm databases, integrating 2470 clinically or experimentally validated interactions between 1373 drugs and 173 microbes, and 2884 verified associations involving 140 microbes and 1720 drugs. To gain deeper insights into microbes, drugs, and related entities, we constructed a comprehensive knowledge graph by integrating information from multiple data sources, including studies by Jiang *et al.* (2023), MEGARes, DrugVirus, NMDC, and HMDB. The final knowledge graph comprises 81 829 entities, 407 270 triples, and 42 relation types. To resolve annotation conflicts across databases, we prioritized associations with higher experimental validation frequency and retained only those supported by at least two independent studies. Missing attributes of microbes or drugs were imputed using relational context from the knowledge graph, such as metabolic pathway membership and involvement in biological processes. Our model framework is illustrated in Fig. 1. To further illustrate the operational mechanism of multi-level contrastive learning in the model, Fig. 2 presents the multi-level contrastive mechanism for predicting potential microbe–drug associations.

To predict potential microbe–drug relationships, we use known microbe–drug interactions and a knowledge graph to identify latent connections. We construct a complex model integrating deep learning for multi-level representation learning. Given $D=\{d_{1},d_{2},\ldots ,d_{n}\}$ as the set of drugs and $M=\{m_{1},m_{2},\ldots ,m_{n}\}$ as the set of microbes, we build a microbe–drug adjacency matrix $A\in R^{M\times N}$, where $A_{i,j}=1$ if there is a known interaction between drug $d_{i}$ and microbe $m_{j}$, and 0 otherwise. Additionally, the knowledge graph G={(h,r,t)|h,t∈E,r∈R} captures relationships between entities E via relations R. Our objective is to learn a function F(m,d|θ) that predicts the potential association score ${\hat{y}}_{m,d}$ between microbes and drugs, using A and G. The model is optimized to minimize the cross-entropy loss function:

(1) $$L_{\mathrm{pred}}=-\frac{1}{\left|D\right|}\sum_{(m,d)\in D}\left[A[m,d]\;\log\;{\hat{y}}_{m,d}+(1-A[m,d])\;\log\;\left(1-{\hat{y}}_{m,d}\right)\right]$$

### 2.1 Local graph construction

We construct the local graph by capturing both direct and indirect relationships between microbes and drugs, focusing on first-order interactions and their related knowledge graph information. First, we extract collaborative filtering signals between microbes m and drugs d from matrix A, defining the direct interaction sets as $D_{u}=\{u|A_{u}=1\},u=(m,d)$, These interactions provide the foundation for subsequent graph construction. Next, we map the collaborative filtering signals to the entity space in the knowledge graph through an entity alignment process, constructing the initial node sets for the local graph. The initial entity sets $E_{d,L}^{0}$ are defined as:

(2) Eu,L0={e|(u,e)∈A,u∈{u|Au=1}}#

This process successfully maps the signals to entities in the knowledge graph. Finally, we propagate the initial entity sets through the structural relationships in the knowledge graph to generate multi-layered local graph structures. Specifically, the propagation process for microbes and drugs is as follows:

(3) Su,Ll={(h,r,t)|(h, r, t)∈G, h∈Eu,Ll-1,l=1,…,L#

where $E_{u,L}^{l-1}$ represents the entity set after propagation at layer l-1. The final entity set at layer L is:

(4) Eu,Ll={t|(h,r,t)∈Su,Ll}#

### 2.2 Non-local graph construction

The non-local graph incorporates high-order collaborative filtering (CF) signals (e.g. microbe–drug–disease co-occurrence) and additional external knowledge graph (KG) facts to enhance the representations of microbes and drugs. The construction process is similar to that of the local graph but extends the information by aligning high-order CF drugs with the knowledge graph and propagating through the KG.

First, high-order drugs are obtained by propagating through the microbe–drug interactions. Specifically, the high-order drug set $D_{p}$ for microbe m is defined as:

(5) Dp={dp|u∈Msim,Amdp=1}#

Similarly, the set of similar microbes $M_{\mathrm{sim}}$ is defined by drugs interacted with and the propagation of similarity:

(6) Msim={usim|d∈{d|A=1} and Amsimd=1}#

Next, these high-order drugs are aligned with entities in the knowledge graph to construct the initial entity sets. The initial entity sets for microbes and drugs are defined as:

(7) Eu,N0={e|(dp,e)∈A,dp∈Dp}#

Through propagation in the knowledge graph, we further construct the non-local graph. The propagated triple sets are represented as:

(8) Su,Nl={(h,r,t)|(h, r, t)∈G, h∈Eu,Nl-1,l=1,…,L#

Finally, after multiple layers of propagation, the entity set $E_{u,N}^{L}$ at layer L reveals more complex latent relationships between microbes and drugs:

(9) Eu,Nl={t|(h,r,t)∈Su,Nl}#

Through recursive propagation, the non-local graph captures complex indirect relationships, providing richer semantic information for microbe–drug association tasks.

### 2.3 Embedding representation

#### 2.3.1 Pre-training embedding

To enhance the initial expressiveness of the model, we pretrain the knowledge graph using the TransE model to generate embedding representations for entities and relations. TransE maps the triples (h,r,t) into a low-dimensional space and uses an additive structure to model the relationships between the triples:

(10) h+r≈t,∀(h,r,t)∈T#

where h, r and t represent the embeddings of the head entity, relation, and tail entity, respectively. To optimize the model, TransE employs a negative sampling strategy to generate false triples, which are used to train the model to distinguish between true and false relationships. The loss function is as follows:

(11) LTransE=∑(h,r,t)∈Gmax⁡(0,γ+f(h′,r,t′)-f(h,r,t))#

Through this training process, the model effectively initializes the embedding representations, enhancing its expressiveness and accelerating convergence.

#### 2.3.2 Graph encoding

To better integrate information from both local and non-local graphs, we adopt a multi-head attention mechanism to compute the weighted representation of each triple. In each layer, the embeddings of the head entity and relation are concatenated to form the query vector $q_{i}$, while the embedding of the tail entity serves as both the key $k_{i}$ and value $v_{i}$:

(12) qi=[hi//ri],ki=ti,vi=ti#

The similarity between the query and key vectors is computed, resulting in the attention weights $\alpha_{\mathrm{ij}}^{j}$:

(13) αijj=softmax(qij·(kij)Tdh)#

Using these weights, we aggregate the tail entity information, yielding the output $O_{i}^{j}$ for each attention head. Finally, the outputs from all attention heads are concatenated and a linear transformation is applied to obtain the final embedding representation:

(14) El=Concat(O1,O2,…,OH)WO#

This approach effectively captures the complex relationships between entities, enhancing the richness of the embedding representations and providing more powerful feature representations for subsequent tasks.

### 2.4 Contrastive learning

In the task of predicting potential associations between microbes and drugs, the core objective of constructing a non-local graph is to capture high-order interaction relationships between microbes and drugs, and to extend these relationships by incorporating external knowledge graph information. This approach allows the non-local graph to help capture latent connections and contextual information between microbes and drugs. The non-local graph is primarily constructed through the propagation of high-order collaborative filtering (CF) signals and knowledge graph (KG) information to establish relationships between microbes and drugs. To construct the non-local graph, we first obtain high-order information by propagating the interaction relationships between microbes and drugs. The high-order drug set for a microbe m is represented as:

(15) $$L_{\mathrm{Intra},k^{+},\{k^{-}\}}^{M}=\sum_{m\in M}-\log\frac{\sum_{k\in K}e^{\left(\left(E_{m,L}^{\left(0\right)}\cdot \frac{E_{m,L}^{\left(k^{+}\right)}}{\tau }\right)\right)}}{\sum_{k\in K}e^{\left(\left(E_{m,L}^{\left(0\right)}\cdot \frac{E_{m,L}^{\left(k^{+}\right)}}{\tau }\right)\right)}+\sum_{k^{-}\notin K}e^{\left(\left(E_{m,L}^{\left(0\right)}\cdot \frac{E_{m,L}^{\left(k^{-}\right)}}{\tau }\right)\right)}}+\sum_{m\in M}-\log\frac{\sum_{l\in L}e^{\left(\left(E_{m,N}^{\left(0\right)}\cdot \frac{E_{m,N}^{\left(k^{+}\right)}}{\tau }\right)\right)}}{\sum_{k\in K}e^{\left(\left(E_{m,N}^{\left(0\right)}\cdot \frac{E_{m,N}^{\left(k^{+}\right)}}{\tau }\right)\right)}+\sum_{k^{-}\notin K}e^{\left(\left(E_{m,N}^{\left(0\right)}\cdot \frac{E_{m,N}^{\left(k^{-}\right)}}{\tau }\right)\right)}}$$

where $E_{m,L}^{0}$ represents the embedding of microorganism m in the initial layer (layer 0) of the local graph. $E_{m,L}^{\left(k\right)}$ denotes the embedding of microorganism m in the kth layer of the local graph for the positive sample, defined as the embedding of a layer adjacent to the current sample. On the other hand, $E_{m,L}^{\left(k-\right)}$ indicates the embedding in the kth layer of the local graph for the negative sample, defined as the embedding of a randomly selected, non-adjacent layer within the same graph. Similarly, $E_{m,N}^{0}$ is the embedding in the initial layer of the non-local graph, $E_{m,N}^{\left(k\right)}$ is the embedding in the kth layer of the non-local graph for the positive sample, and $E_{m,N}^{\left(k-\right)}$ is the embedding in the kth layer of the non-local graph for the negative sample. The temperature parameter τ controls the sharpness of the softmax distribution. It is chosen empirically, following standard practice in contrastive learning to balance the impact of positive and negative samples. In this work, we set τ = 0.1, as it has been shown in previous studies to yield optimal results in similar settings (Chen *et al.* 2020). This loss reflects intra-graph contrastive learning, where adjacent layers in the same graph are treated as positive samples, and randomly selected non-adjacent layers are treated as negatives.

Ultimately, the total intra-graph interactive contrastive loss is the sum of the contrastive losses for microbes and drugs:

(16) LIntra=LIntra,k+,{k-}M+LIntra,k+,{k-}N#

This method effectively combines collaborative filtering signals and knowledge graph information, enhancing the prediction accuracy of potential associations between microbes and drugs.

While intra-graph contrastive learning can achieve coherent utilization of information within a single graph, the noisy nature of the non-local graph poses challenges for integrating information from local and non-local graphs. To reduce noise and extract meaningful information, we propose an inter-graph contrastive learning approach. In this method, any layer in the local graph serves as an anchor, with the corresponding layer in the non-local graph acting as positive sample pairs, and other layers as negative sample pairs. The contrastive loss for microbes is formulated as:

(17) $$L_{\mathrm{Inter},k^{+},\{k^{-}\}}^{M}=\sum_{m\in M}-\log\frac{\sum_{k\in K}e^{\left(\left(E_{m,L}^{\left(k^{+}\right)}\cdot \frac{E_{m,N}^{\left(k^{+}\right)}}{\tau }\right)\right)}}{\sum_{k\in K}e^{\left(\left(E_{m,L}^{\left(k^{+}\right)}\cdot \frac{E_{m,N}^{\left(k^{+}\right)}}{\tau }\right)\right)}+\sum_{k^{-}\notin K}e^{\left(\left(E_{m,L}^{\left(k^{+}\right)}\cdot \frac{E_{m,N}^{\left(k^{-}\right)}}{\tau }\right)\right)}}$$

This loss captures inter-graph contrast, aligning representations of the same entity across local and non-local graphs to improve consistency and reduce graph-specific noise. The inter-graph contrastive loss is the sum of the contrastive losses for microbes and drugs:

(18) LInter=LInter,k+,{k-}M+LInter,k+,{k-}D#

Through this method, the local graph and the non-local graph can jointly extract useful information and reduce noise, thereby improving the accuracy of predicting associations between microbes and drugs.

### 2.5 The final association prediction

Finally, we concatenate the multi-layer embeddings from both the local and non-local graphs to form the final representation:

(19) em=Concat(Em,L0,…,Em,LL,Em,N0,…,Em,NL)#

 

(20) ed=Concat(Ed,L0,…,Ed,LL,Ed,N0,…,Ed,NL)#

Then, we obtain the prediction results by calculating the matching score between the microbe m and the drug d:

(21) y^m,d=σ(emTed)#

To evaluate the effectiveness of the microbe–drug potential association prediction model, we implemented 5-fold cross-validation. The known associations were randomly divided into five subsets, and in each round of validation, one subset was used as the test set (containing an equal number of positive and negative samples), while the remaining subsets constituted the training set.

(22) TPR=TPTP+FN#

 

(23) FPR=FPTN+FP#

The model predicts scores for the test samples and compares them to a preset threshold to determine the results. Performance metrics include the area under the ROC curve (AUC) and the area under the PR curve (AUPR), which are used to evaluate the classification performance across different thresholds and the prediction efficacy in the context of imbalanced data, respectively.

By averaging the results of the 5-fold cross-validation, we can effectively reduce the potential impact of randomness due to the arbitrary division of the dataset, ensuring the reliability and robustness of the experimental results. All experiments were conducted on a high-performance computing server equipped with NVIDIA A100 GPUs. The model was trained using the Adam optimizer with a learning rate of 0.1, a batch size of 64, and for 200 epochs. An early stopping mechanism was applied, terminating training if the validation AUC did not improve for 10 consecutive epochs. The random seed was fixed at 42 to ensure reproducibility.

## 3 Results

### 3.1 Baseline comparison

We conducted 5-fold cross-validation on the MDAD and aBiofilm datasets to compare KGCLMDA with five baseline methods:

- GCNMDA (Long *et al.* 2020): Combines GCN and CRF for better neighborhood aggregation.
- GSAMDA (Tan *et al.* 2022): Uses GAT for node features and SAE for attributes to improve predictions.
- MDASAE (Fan *et al.* 2023): Combines attention mechanisms and autoencoders to enhance feature extraction.
- LRLSHMDA (Wang *et al.* 2017): Uses Gaussian kernel similarity and LRLS for topological feature mining.
- NTSHMDA (Luo and Long 2018): Uses RWR and network similarity to predict complex associations.

The results in Tables 1–3 show that KGCLMDA outperforms the other methods in AUC and AUPR, improving AUC by 0.0305 (MDAD), 0.0269 (aBiofilm), and 0.0257(DrugVirus), and AUPR by 0.073 (MDAD), 0.0370 (aBiofilm), and 0.0502 (DrugVirus).

**Table 1.** Performance comparison of baseline methods and KGGCLMDA on MDAD.

Methods	AUC	AUPR
GCNMDA	0.9282	0.9126
GSAMDA	0.9488	0.4520
MDASAE	0.9565	0.3655
LRLSHMDA	0.9400	0.6351
NTSHMDA	0.8484	0.1374
KGCLMDA	0.9870	0.9856

**Table 2.** Performance comparison of baseline methods and KGGCLMDA on aBiofilm.

Methods	AUC	AUPR
GCNMDA	0.9549	0.9499
GSAMDA	0.9353	0.9317
MDASAE	0.9613	0.9536
LRLSHMDA	0.9512	0.6796
NTSHMDA	0.8629	0.1407
KGCLMDA	0.9882	0.9869

**Table 3.** Performance comparison of baseline methods and KGGCLMDA on DrugVirus.

Methods	AUC	AUPR
GCNMDA	0.9330	0.9236
GSAMDA	0.9281	0.9137
MDASAE	0.9489	0.9312
LRLSHMDA	0.9356	0.6274
NTSHMDA	0.8357	0.1307
KGCLMDA	0.9746	0.9738

**Figure 3.**
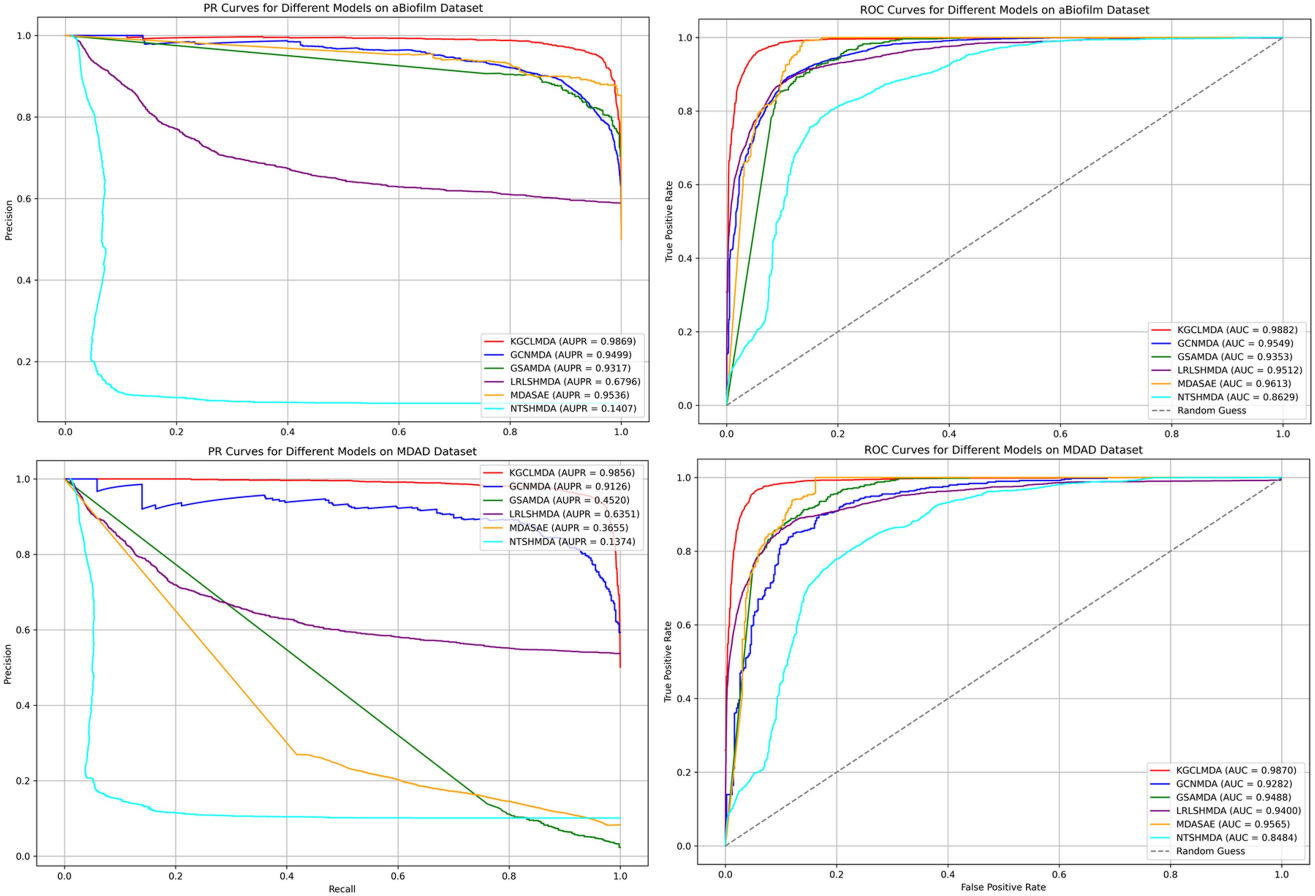
Comparison of PR and ROC curves for different models on the aBiofilm and MDAD datasets.

Figure 3 shows the PR and ROC curves for each method, and the results demonstrate that KGCLMDA outperforms the other methods on both datasets, achieving the highest AUPR and AUC values, highlighting its superiority in microbe–drug association prediction.

### 3.2 Hyperparameter sensitivity analysis

In the hyperparameter sensitivity analysis, we examined how model performance changes with variations in the number of knowledge graph propagation n-layers, learning rate (lr), and contrastive learning hyperparameters (ssl_temp, ssl_reg, ssl_reg_inter). The results indicate that n-layers and lr are critical factors, and appropriate settings for ssl_temp and regularization improve model stability and prediction performance.

**Figure 4.**
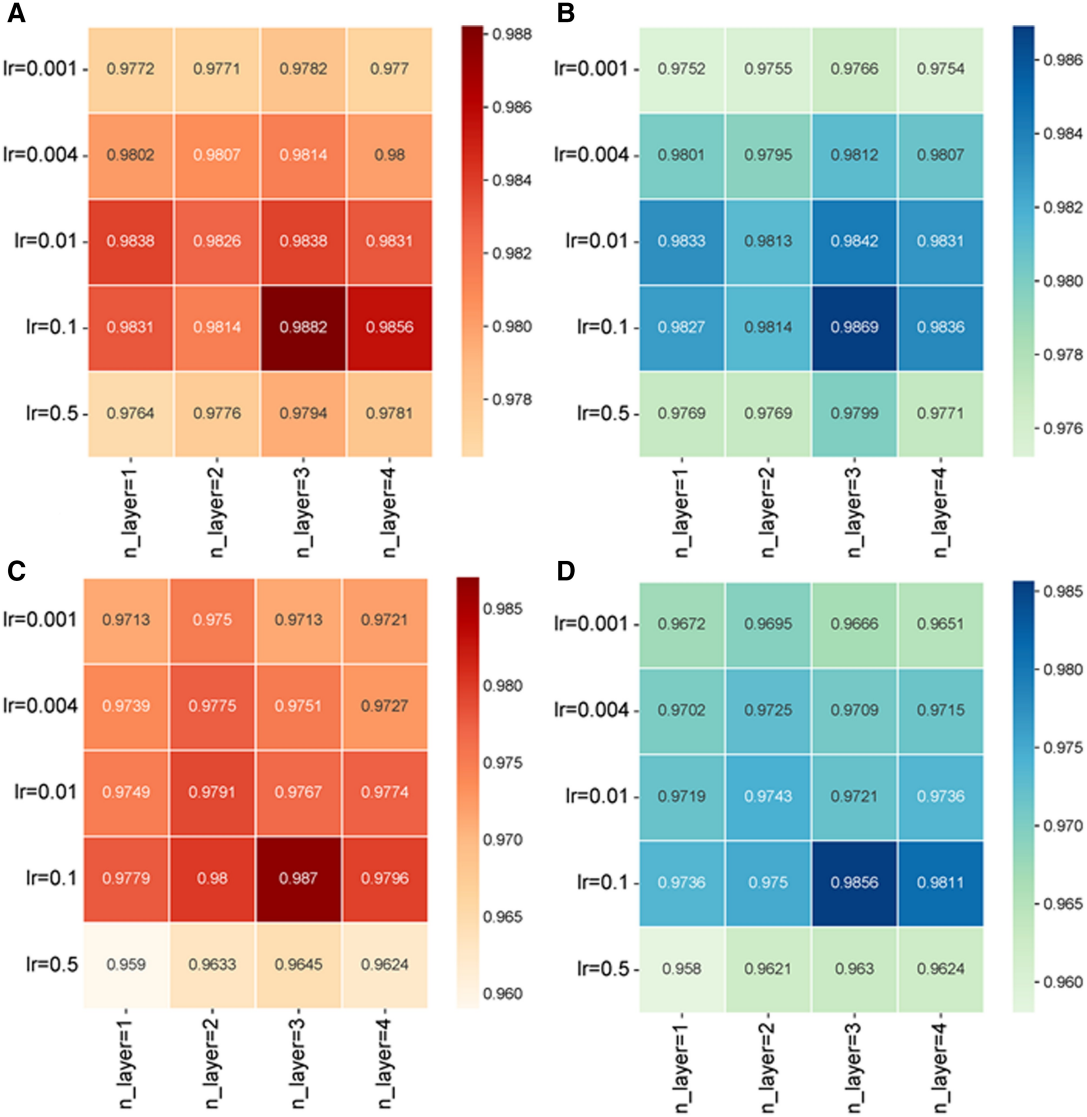
Performance evaluation of the model. (A) AUPR values of the model on the aBiofillm. (B) AUC values of the model on the aBiofilm. (C) AUPR values of the model on the MDAD. (D) AUC values of the model on the MDAD.

**Figure 5.**
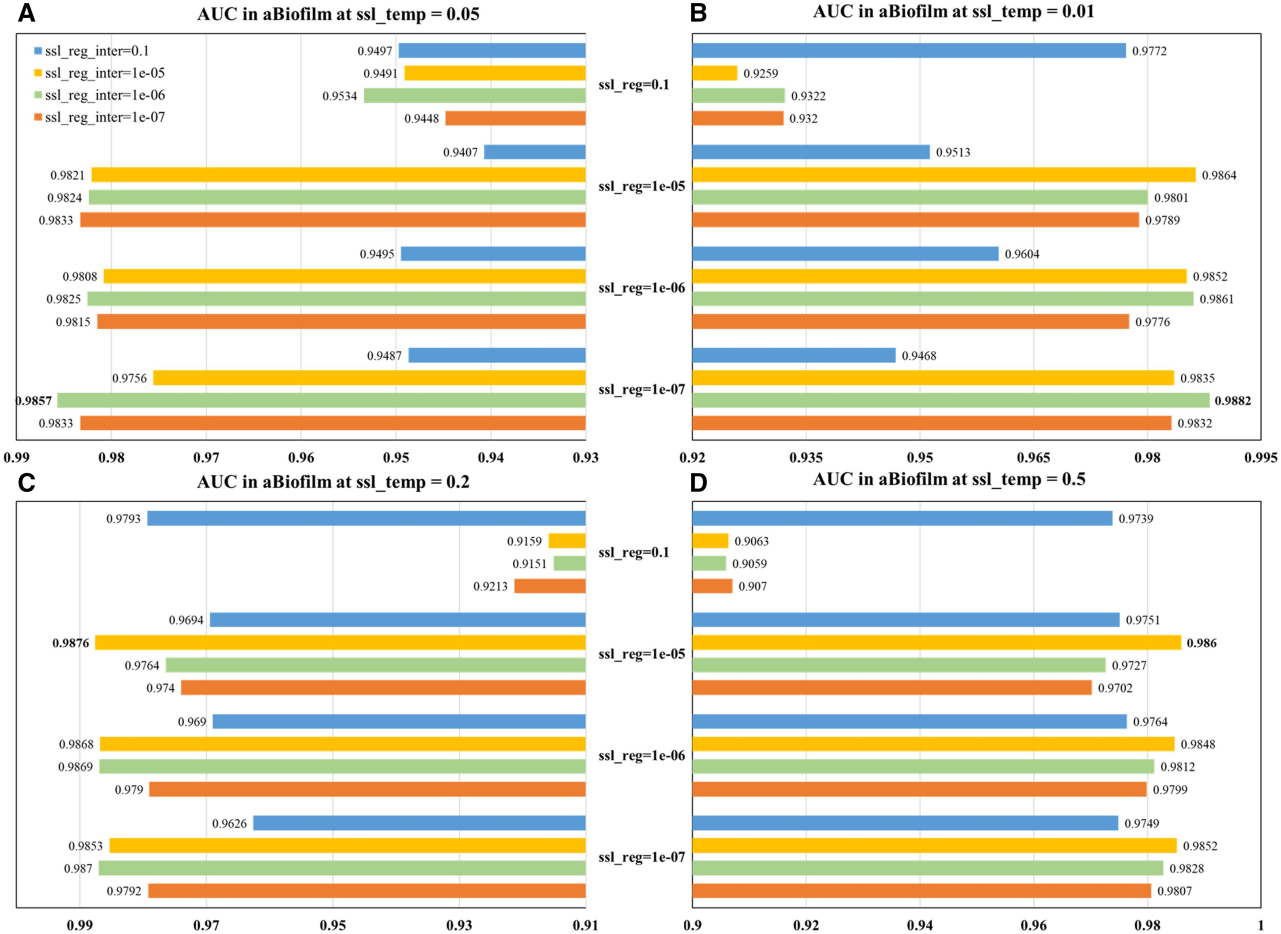
AUC performance evaluation of the model on aBiofilm dataset under different hyperparameter settings. Subfigures A, B, C, and D respectively present results when ssl_temp is set to 0.05, 0.01, 0.2, and 0.5.

**Figure 6.**
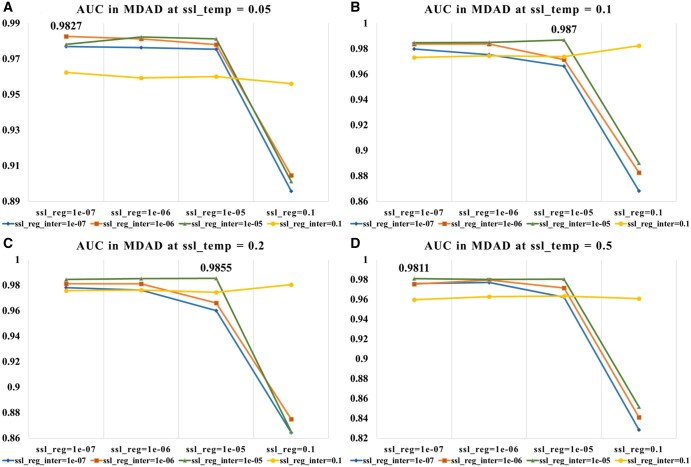
AUC performance evaluation of the model on MDAD dataset under different hyperparameter settings. Subfigures A, B, C, and D respectively present results when ssl_temp is set to 0.05, 0.1, 0.2, and 0.5.

**Figure 7.**
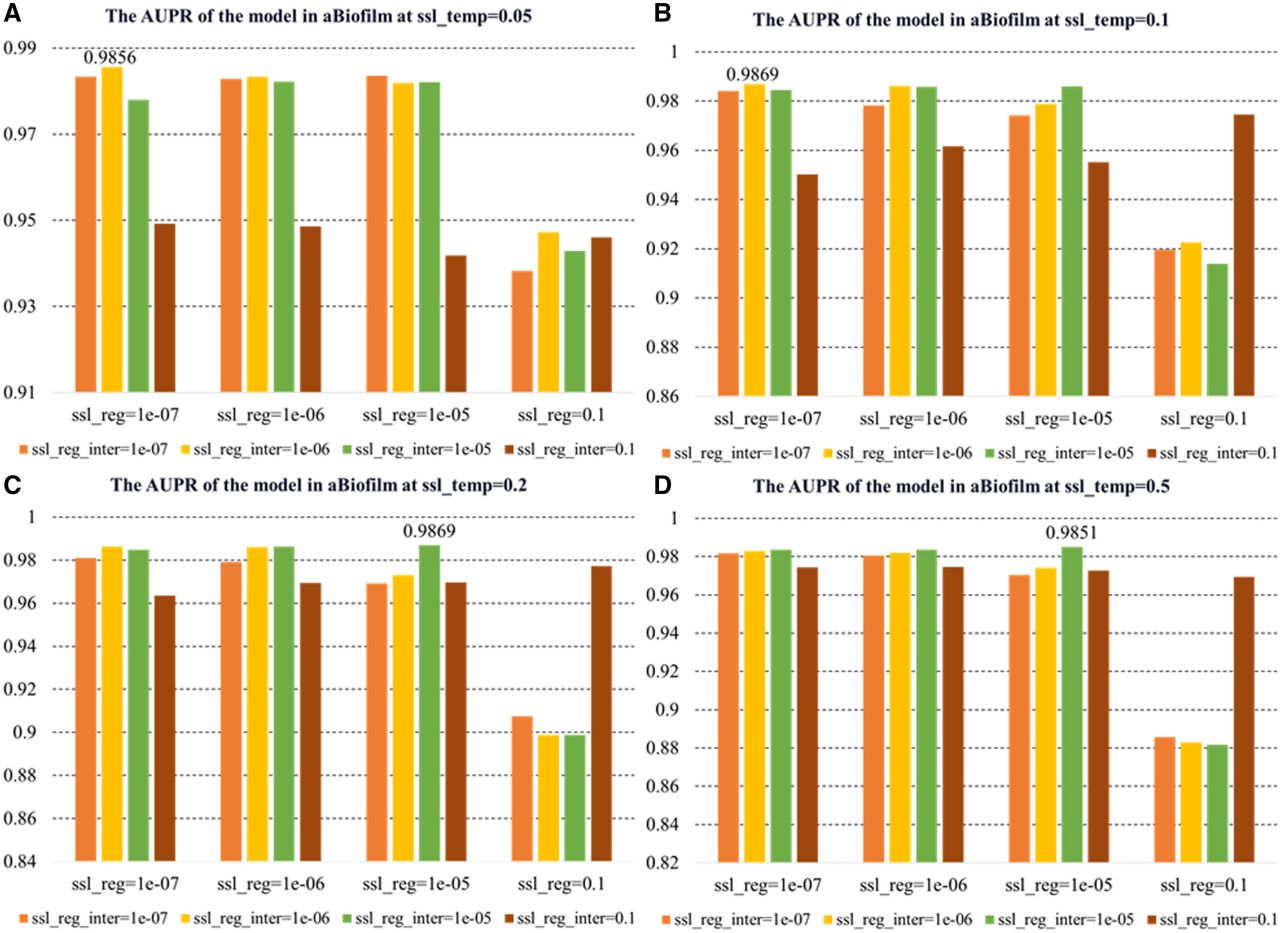
AUPR performance evaluation of the model on aBiofilm dataset under different hyperparameter settings. Subfigures A, B, C, and D respectively present results when ssl_temp is set to 0.05, 0.1, 0.2, and 0.5.

**Figure 8.**
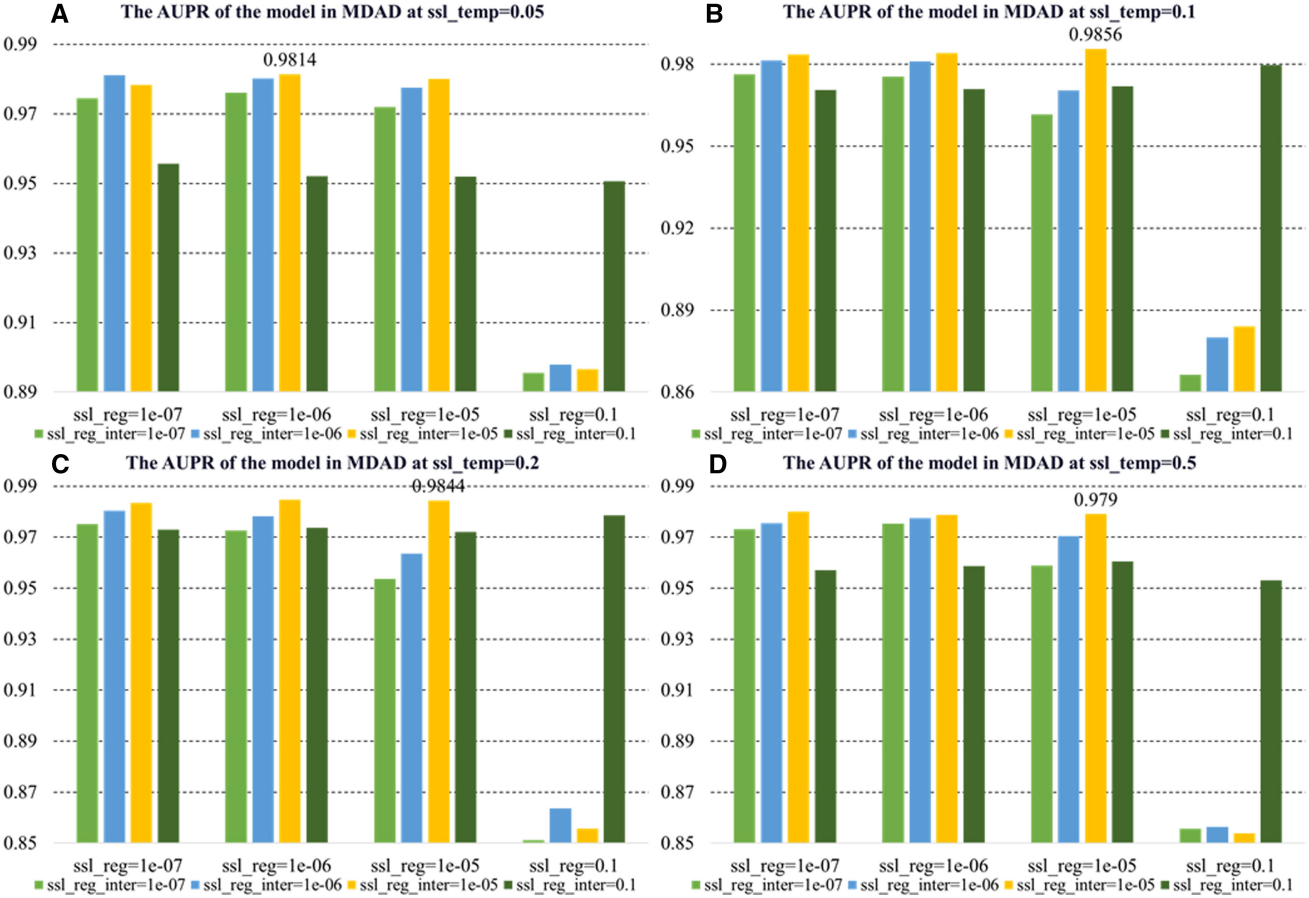
AUPR performance evaluation of the model on MDAD dataset under different hyperparameter settings. Subfigures A, B, C, and D respectively present results when ssl_temp is set to 0.05, 0.1, 0.2, and 0.5.

As shown in Fig. 4, KGCLMDA performs best on both the MDAD and aBiofilm datasets when the learning rate lr is set to 0.1 and n-layers is set to 3. Figures 5 and 6 show that the model achieves optimal performance on the aBiofilm and MDAD datasets when the hyperparameters are set to {ssl_temp = 0.1, ssl_reg = 1e-07, ssl_reg_inter = 1e-06} and {ssl_temp = 0.1, ssl_reg = 1e-05, ssl_reg_inter = 1e-05}, respectively. Figures 7 and 8 further confirm these optimal hyperparameter settings for AUPR performance.

### 3.3 Ablation study

**Figure 9.**
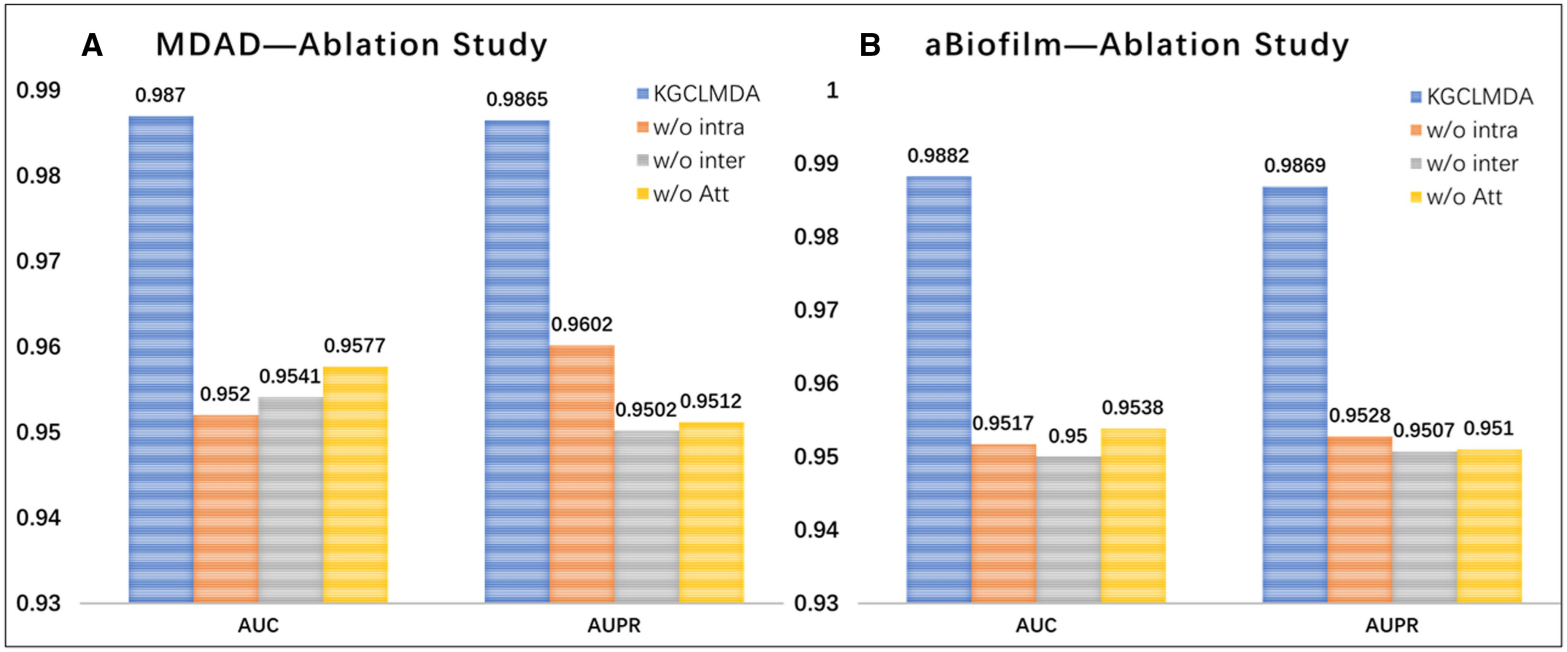
Ablation study. Subfigures A and B respectively present the ablation study results on MDAD and aBiofilm datasets.

We conducted ablation studies to evaluate the impact of key components on model performance by modifying the model input, structure, or dataset. We created three variants by disabling one component each: (i) the first variant removes intra-layer contrastive learning (w/o intra), (ii) the second variant removes inter-layer contrastive learning (w/o inter), and (iii) the third variant removes the multi-layer attention mechanism (w/o Att). As shown in Fig. 9, KGCLMDA outperforms all variants, demonstrating that the combination of all components contributes to its superior performance.

### 3.4 Case study

To demonstrate the predictive performance of KGCLMDA, we conducted case studies on two drugs and two microorganisms. We selected the top 20 microorganisms and drugs predicted by KGCLMDA based on the MDAD database and verified their presence in the literature. The first drug is Moxifloxacin, a broad-spectrum fluoroquinolone effective against Gram-positive bacteria and anaerobes (Barman Balfour and Wiseman 1999), relevant in antibiotic-resistant infections (Loyola-Rodriguez *et al.* 2018). The second drug is Ciprofloxacin, a widely used fluoroquinolone that inhibits bacterial DNA gyrase and treats various infections (Campoli-Richards *et al.* 1988, Sharma *et al.* 2010). Tables 4 and 5 show that 17 and 18 of the top 20 predicted microorganisms for Moxifloxacin and Ciprofloxacin, respectively, are validated by the literature.

**Table 4.** Top 20 predicted microbe candidates related to Moxifloxacin.

Microbe	Evidence	Microbe	Evidence
Pseudomonas aeruginosa	PMID:31643179	Vibrio harveyi	NA
Escherichia coli	PMID:36250047	HIV-1	PMID:18441333
Staphylococcus aureus	PMID:31689174	Haemophilus influenzae	NA
Streptococcus mutans	PMID:29392681	Actinomyces oris	PMID:26538502
Candida albicans	PMID:12121916	Burkholderia cenocepacia	PMID:33120688
Staphylococcus epidermis	PMID:11249827	Enteric bacteria and other eubacteria	NA
Staphylococcus epidermidis	PMID:31516359	Streptococcus pneumoniae	PMID:31542319
Bacillus subtilis	PMID:30036828	Listeria monocytogenes	PMID:28739228
Enterococcus faecalis	PMID:31763048	Burkholderia pseudomallei	PMID:15731198
Salmonella enterica	PMID:22151215	Stenotrophomonas maltophilia	PMID:31748318

**Table 5.** Top 20 predicted microbe candidates related to Ciprofloxacin.

Microbe	Evidence	Microbe	Evidence
*Pseudomonas aeruginosa*	PMID:30605076	Haemophilus influenzae	NA
*Staphylococcus aureus*	PMID:36499677	Salmonella enterica	PMID:26933017
*Candida albicans*	PMID:35404123	Streptococcus sanguis	PMID:11347679
*Streptococcus mutans*	PMID:30468214	Enteric bacteria and other eubacteria	PMID:274364661
*Staphylococcus epidermis*	PMID:10632381	Serratia marcescens	PMID:23751969
*Escherichia coli*	PMID:2325984	HIV-1	PMID:9566552
*Staphylococcus epidermidis*	PMID:28481197	Actinomyces oris	NA
*Vibrio harveyi*	PMID:27247095	Listeria monocytogenes	PMID:28355096
*Bacillus subtilis*	PMID:33218776	Burkholderia multivorans	PMID:19633000
*Enterococcus faecalis*	PMID:27790716	Streptococcus epidermidis	PMID:9111541

## 4 Conclusions

This paper proposes a novel method for predicting microbe–drug associations based on knowledge graphs and contrastive learning, named KGCLMDA (Knowledge Graph and Contrastive Learning Model). The method aims to predict potential microbe–drug associations by leveraging known microbe–drug interaction data and external knowledge graphs. The core objective of the research is to address the challenges of data scarcity, imbalanced information utilization, and insufficient knowledge extraction in traditional methods. Our model combines knowledge graph embedding and contrastive learning techniques, preprocessing and modeling the embedding representations of microbes and drugs, and integrating both local and non-local graph structures to effectively capture the direct and indirect relationships between microbes and drugs. Experimental results show that KGCLMDA significantly outperforms existing methods in metrics such as AUC and AUPR. This study provides an effective solution for microbe–drug association prediction and contributes to the fields of biomedical research and precision medicine.

Our approach constructs both local and non-local graphs to capture direct interactions between microbes and drugs, as well as indirect relationships related to microbial metabolic functions or drug chemical properties. First, we preprocess microbes and drugs using the TransE model, embedding them into a low-dimensional space to capture latent semantic relationships. Next, we incorporate a multi-head attention mechanism during graph encoding to compute weighted representations for each triple, enhancing the model’s ability to capture complex relationships. The contrastive learning mechanism is introduced to optimize embedding representations, performing contrastive learning both within and between layers, improving the learning of local relationships and the integration of non-local information. The contributions of this work are summarized as follows:

- The KGCLMDA model is proposed, which combines knowledge graphs and contrastive learning for microbe–drug association prediction.
- Local and non-local graphs are constructed to capture direct and indirect relationships between microbes and drugs.
- A multi-level contrastive learning mechanism is integrated to improve the model’s predictive performance in data-sparse and noisy environments.
- Experiments show that KGCLMDA exhibits superior performance in the task of microbe–drug association prediction and has practical application feasibility.

Although KGCLMDA has achieved promising results, there is still room for improvement. Future research can explore dynamic knowledge graphs to optimize prediction results as new data becomes available. Additionally, incorporating temporal information or patient-specific data could further enhance prediction accuracy, supporting personalized drug recommendations. This model offers new insights into microbe–drug interaction prediction, laying the foundation for applications in precision medicine, drug repositioning, and microbiome research, and is expected to drive the development of personalized healthcare.
